# Supplementary material for: Winter associations predict social and extra-pair mating patterns in a wild songbird
Source: Proc Biol Sci. 2020 Feb 19;287(1921):20192606. doi: 10.1098/rspb.2019.2606 (PMC7062020; doi:10.1098/rspb.2019.2606)
Supplement: Results of additional analyses [file rspb20192606supp1.docx]

**Supplementary material for:**

**Winter associations predict social and extra-pair mating patterns in a wild songbird**

Kristina B. Beck^1 *^, Damien R. Farine^2,3,4^, Bart Kempenaers^1^

^1^ Department of Behavioural Ecology and Evolutionary Genetics, Max Planck Institute for Ornithology, Seewiesen, Germany

^2^ Department of Collective Behaviour, Max Planck Institute of Animal Behavior, Konstanz, Germany

^3^ Department of Biology, University of Konstanz, Germany

^4^ Centre for the Advanced Study of Collective Behaviour, University of Konstanz, Germany

^*^ Corresponding author

Proceedings of the royal society B – Biological Sciences

DOI: 10.1098/rspb.2019.2606

**Figures**

**
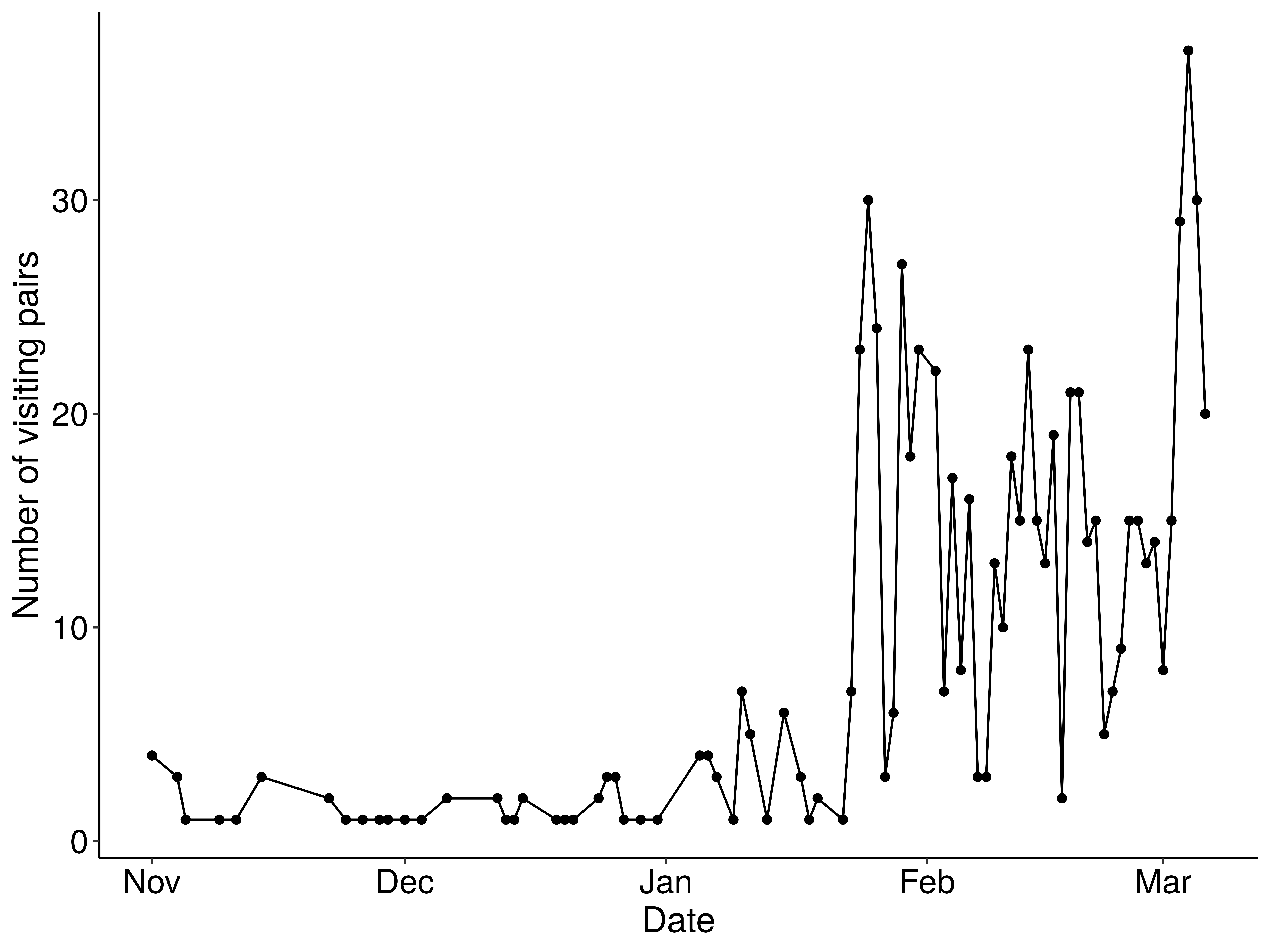
**

**Figure S1.** Plot showing the number of unique pairs that visited a nestbox together during the non-breeding phase (November – mid March). The y-axis represents the number of pairs that visited a nestbox and the x-axis represents the date on which the pair visited.

**
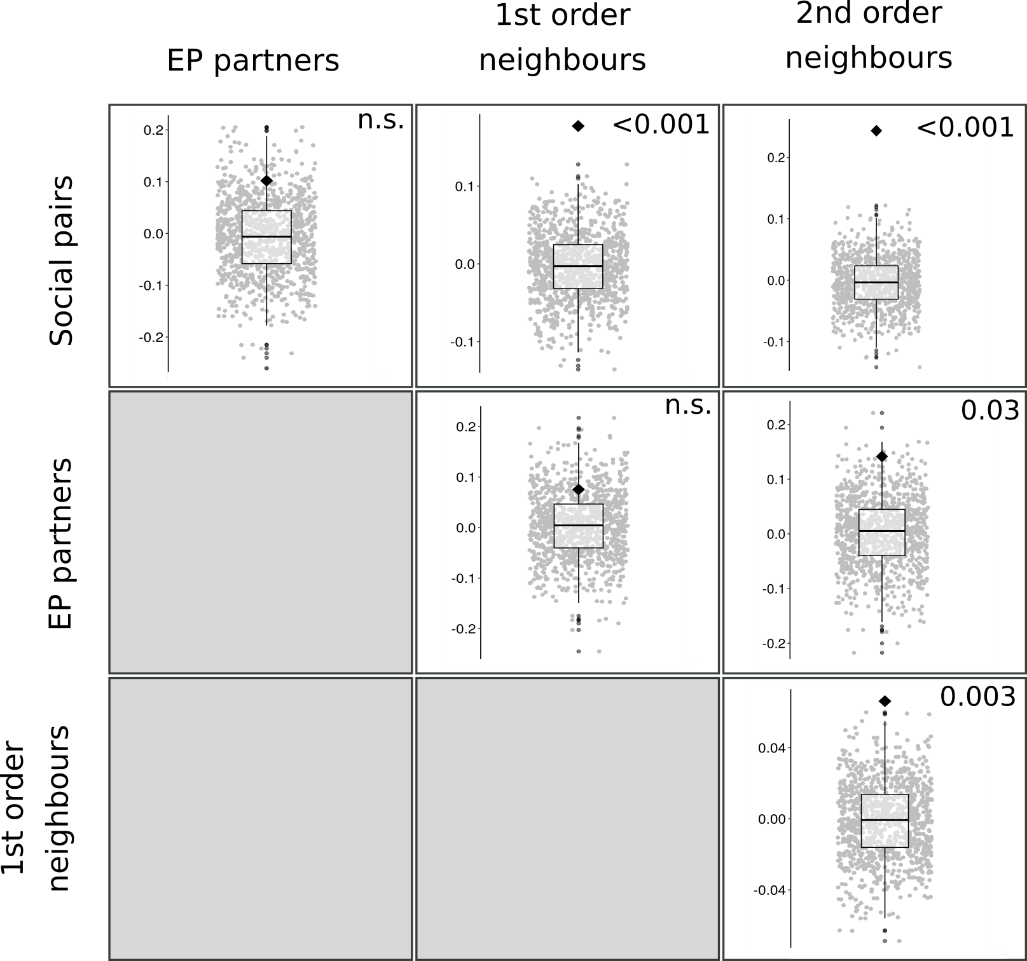
**

**Figure S2.** Figure showing the calculated difference in the mean association strength between different relationship categories (i.e., social pairs - extra-pair (EP) partners, social pairs – 1^st^ order neighbours, social pairs – 2^nd^ order neighbours, etc.). Boxplots and grey points represent the calculated differences between relationship categories generated from 1000 permutations. Boxplots show the minimum values, lower quartile, median, upper quartile, maximum values and outliers (indicated as black dots). The black diamonds represents the difference calculated from the observed data. The p value generated from the randomizations is shown for every relationship comparison in the upper right corner.

**
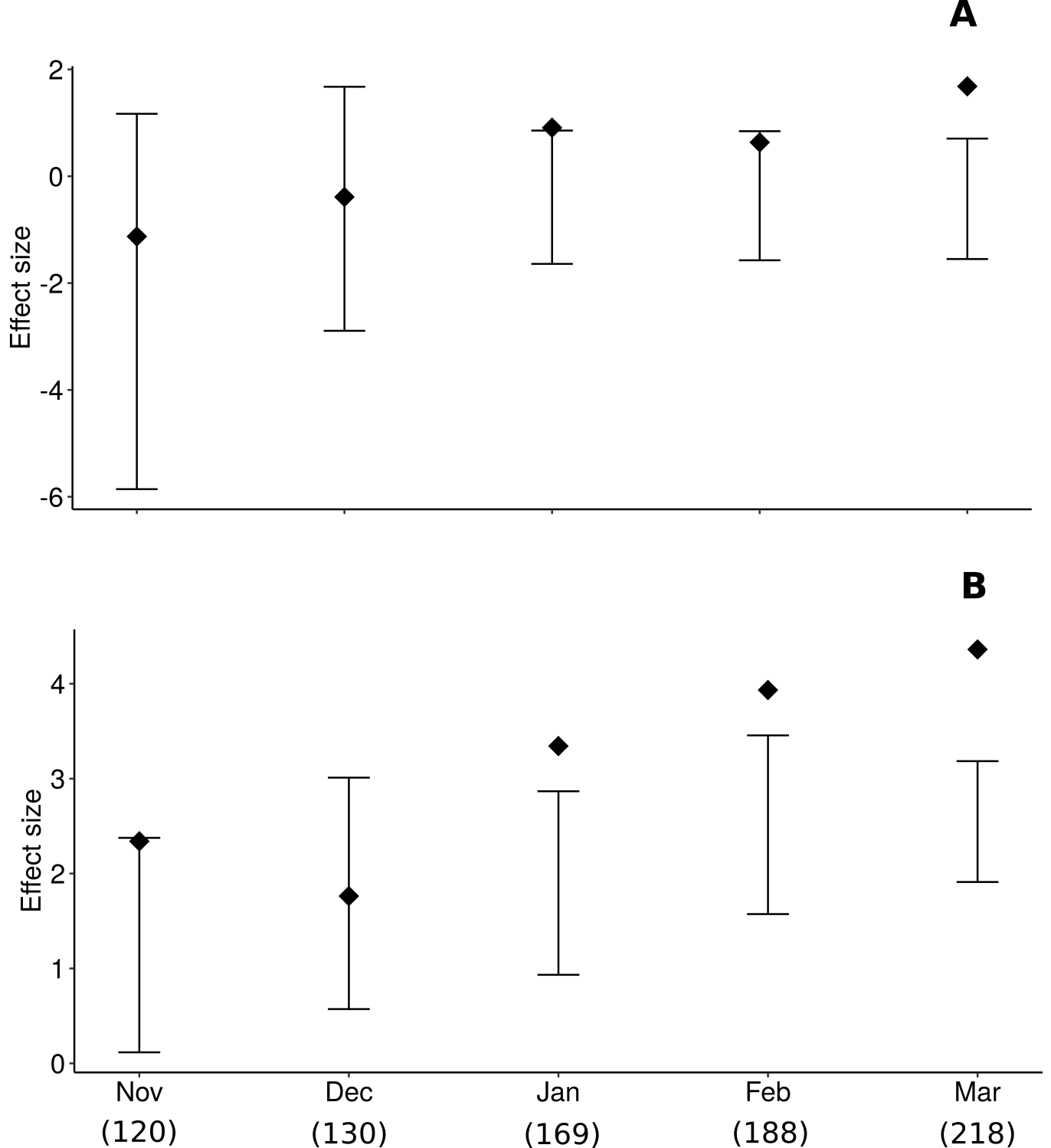
**

**Figure S3.** Effect sizes of the winter association strength during each month before breeding started on the likelihood to become extra-pair partners (A) or social (breeding) pairs (B). Bars indicate the 95% distribution range of the effect sizes generated from random sampling (permutation test); diamonds indicate the observed effect size. Numbers below each month indicate the sample sizes, i.e. the total number of individuals.


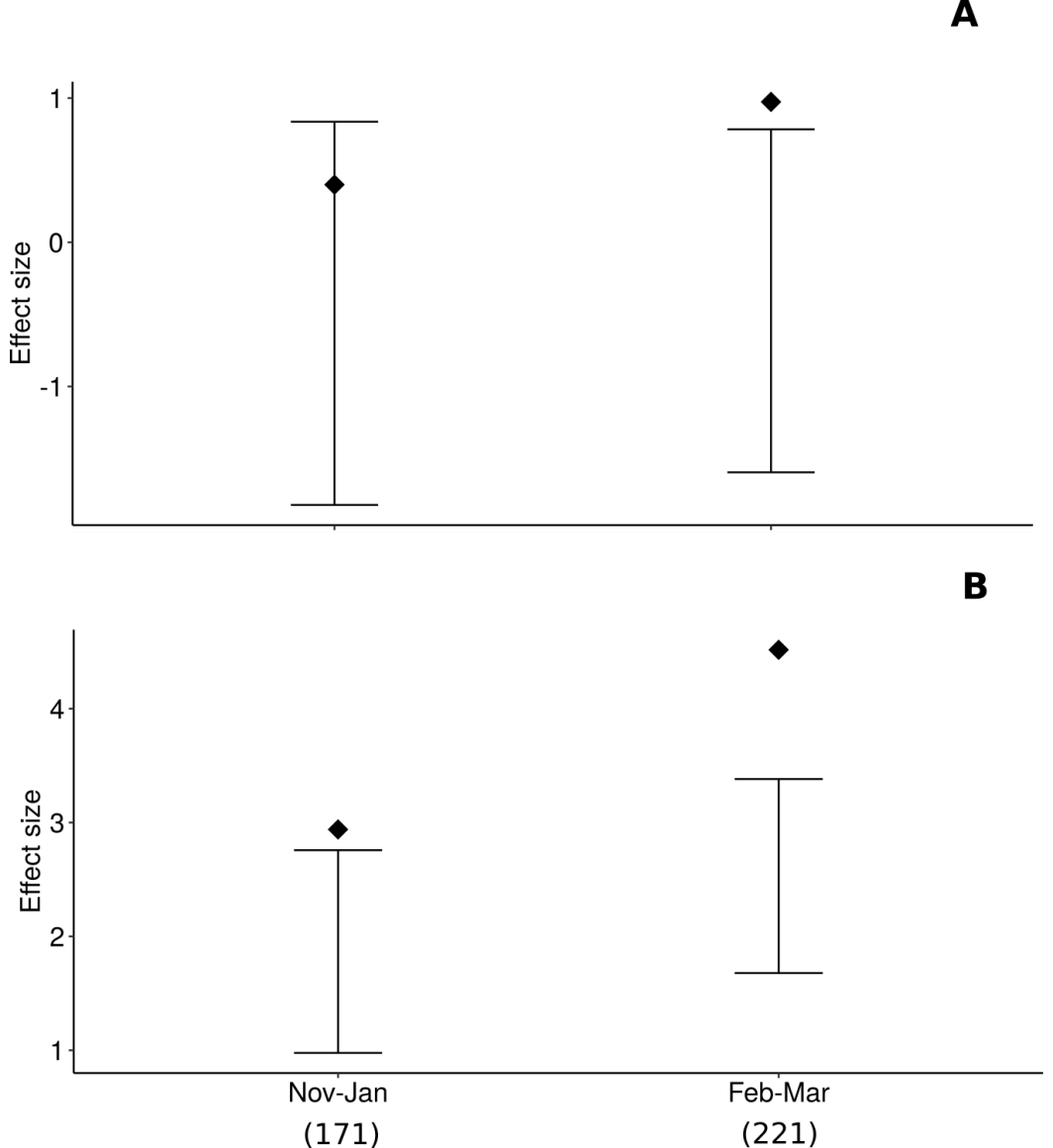


**Figure S4.** Effect sizes of the winter association strength during an early (November-January) and late (February-March) period before breeding started on the likelihood to become extra-pair partners (A) or social (breeding) pairs (B). Bars indicate the 95% distribution range of the effect sizes generated from random sampling (permutation test); diamonds indicate the observed effect size. P values inferred from the permutation tests for the early period: Extra-pair partners=0.44, Social pairs=0.02; late period: Extra-pair partners=0.03, Social pairs<0.001. Numbers below the time period indicate the sample sizes, i.e. the total number of individuals.

**Tables**

Table S1-S2 show the repeated analyses only including individuals which had been equipped with a transponder before the start of the study (N=177, 80% of the 221 individuals which were used for the analyses in the main text, N_Dyads_=7834). For further details on the analyses see the descriptions within the main text.

**Table S1.** Results of the linear network regression model examining the effects of winter association strength and spatial overlap on the breeding proximity of individuals. High overlap in spatial activity and strong winter associations are both associated with breeding closer together. The P-value inferred from the permutation test is shown in italic.

|  | **Estimate** | **P** |
| --- | --- | --- |
| Intercept | 3.42 |  |
| Winter association strength | - 0.22 | *< 0.001* |
| Overlap in spatial activity | - 1.00 | < 0.001 |

**Table S2.** Results of logistic network regression models examining the effect of winter association strength on the likelihood of a female-male dyad to have extra-pair young together. The first model included all neighbourhoods (1^st^ -5^th^ order). The second model included only individuals that ended up as 1^st^ or 2^nd^ order neighbours. P-values inferred from the permutation tests are shown in italic.

|  | **All neighbourhoods** | |  |  | **1^st^ and 2^nd^ order neighbourhoods** | | |
| --- | --- | --- | --- | --- | --- | --- | --- |
|  | **Estimate** | **Exp(b)** | **P** |  | **Estimate** | **Exp(b)** | **P** |
| Intercept | - 6.20 | 0.002 |  |  | - 4.27 | 0.01 |  |
| Neighbourhood order | - 2.36 | 0.09 | < 0.001 |  | - 0.98 | 0.38 | 0.03 |
| Male age* | 0.46 | 1.59 | 0.20 |  | 0.83 | 2.29 | 0.06 |
| Winter association strength | 1.22 | 3.38 | *0.01* |  | 1.08 | 2.94 | *0.03* |
| Box visit† | 0.50 | 1.65 | 0.006 |  | 0.82 | 2.27 | 0.006 |
| Difference in arrival time | 0.62 | 1.85 | 0.16 |  | 0.28 | 1.32 | 0.58 |

*****Adults compared to yearlings.

†Visiting a box together before the start of breeding (compared to no visit).

**Table S3.** Results of logistic network regression models examining the effect of winter association strength on the likelihood of a female-male dyad to have extra-pair young together. The first model included only dyads where at least one individual was unfamiliar to the study site (N_Dyads_=18034). The second model included only dyads where both individuals had been breeding in our study site in 2017 (Dyads familiar from previous season, N_Dyads_=3164). Both models include all neighbourhoods (1^st^ -5^th^ order). P-values inferred from the permutation tests are shown in italic.

|  | **Dyads unfamiliar from previous season** | | |  | **Dyads familiar from previous season** | | |
| --- | --- | --- | --- | --- | --- | --- | --- |
|  | **Estimate** | **Exp(b)** | **P** |  | **Estimate** | **Exp(b)** | **P** |
| Intercept | - 6.83 | 0.001 |  |  | - 5.59 | 0.004 |  |
| Neighbourhood order | - 2.20 | 0.11 | < 0.001 |  | - 2.79 | 0.06 | < 0.001 |
| Male age* | 1.49 | 4.42 | 0.05 |  | ** | | |
| Winter association strength | 0.93 | 2.55 | *0.05* |  | 1.18 | 3.26 | *0.03* |
| Box visit† | 0.38 | 1.48 | 0.12 |  | 0.44 | 1.55 | 0.05 |
| Difference in arrival time | 0.86 | 2.35 | 0.14 |  | 0.51 | 1.67 | 0.32 |

*****Adults compared to yearlings.

†Visiting a box together before the start of breeding (compared to no visit).

** Not applicable as all previously breeding birds are adults.
